# Global application of an unoccupied aerial vehicle photogrammetry protocol for predicting aboveground biomass in non‐forest ecosystems

**DOI:** 10.1002/rse2.228

**Published:** 2021-07-07

**Authors:** Andrew M. Cunliffe, Karen Anderson, Fabio Boschetti, Richard E. Brazier, Hugh A. Graham, Isla H. Myers‐Smith, Thomas Astor, Matthias M. Boer, Leonor G. Calvo, Patrick E. Clark, Michael D. Cramer, Miguel S. Encinas‐Lara, Stephen M. Escarzaga, José M. Fernández‐Guisuraga, Adrian G. Fisher, Kateřina Gdulová, Breahna M. Gillespie, Anne Griebel, Niall P. Hanan, Muhammad S. Hanggito, Stefan Haselberger, Caroline A. Havrilla, Phil Heilman, Wenjie Ji, Jason W. Karl, Mario Kirchhoff, Sabine Kraushaar, Mitchell B. Lyons, Irene Marzolff, Marguerite E. Mauritz, Cameron D. McIntire, Daniel Metzen, Luis A. Méndez‐Barroso, Simon C. Power, Jiří Prošek, Enoc Sanz‐Ablanedo, Katherine J. Sauer, Damian Schulze‐Brüninghoff, Petra Šímová, Stephen Sitch, Julian L. Smit, Caiti M. Steele, Susana Suárez‐Seoane, Sergio A. Vargas, Miguel Villarreal, Fleur Visser, Michael Wachendorf, Hannes Wirnsberger, Robert Wojcikiewicz

**Affiliations:** ^1^ Department of Geography College of Life and Environmental Sciences University of Exeter Exeter UK; ^2^ Environment and Sustainability Institute University of Exeter Penryn UK; ^3^ School of GeoSciences University of Edinburgh Edinburgh UK; ^4^ Grassland Science and Renewable Plant Resources Organic Agricultural Sciences Universität Kassel Witzenhausen D‐37213 Germany; ^5^ Hawkesbury Institute for the Environment Western Sydney University Penrith New South Wales Australia; ^6^ Biodiversity and Environmental Management Department Faculty of Biological and Environmental Sciences University of León León Spain; ^7^ Northwest Watershed Research Center USDA Agricultural Research Service Boise Idaho USA; ^8^ Department of Biological Sciences University of Cape Town Cape Town South Africa; ^9^ Department of Environmental and Water Sciences Instituto Tecnológico de Sonora Ciudad Obregón Sonora Mexico; ^10^ Department of Biological Sciences University of Texas at El Paso El Paso Texas USA; ^11^ Joint Remote Sensing Research Program School of Earth and Environmental Sciences University of Queensland Brisbane Queensland 4072 Australia; ^12^ Faculty of Environmental Sciences Czech University of Life Sciences Prague Kamýcká 129 Praha ‐ Suchdol 165 00 Czechia; ^13^ Biology Department San Diego State University San Diego California USA; ^14^ New Mexico State University Las Cruces New Mexico USA; ^15^ Department of Geography and Regional Research University of Vienna Vienna Austria; ^16^ Department of Biological Sciences Northern Arizona University Flagstaff Arizona USA; ^17^ Southwest Watershed Research Center USDA‐Agricultural Research Service Tucson Arizona USA; ^18^ Department of Forest, Rangeland, and Fire Sciences University of Idaho Moscow Idaho USA; ^19^ Department of Physical Geography Trier University Trier Germany; ^20^ School of Biological, Earth and Environmental Sciences University of New South Wales Penrith New South Wales Australia; ^21^ Department of Physical Geography Goethe University Frankfurt am Main Frankfurt am Main Germany; ^22^ University of New Mexico Albuquerque New Mexico USA; ^23^ Grupo de Investigación en Geomática e Ingeniería Cartográfica (GEOINCA) University of León Ponferrada Spain; ^24^ Department of Natural Resources Sul Ross State University Alpine Texas USA; ^25^ Geomatics Division School of Architecture, Planning and Geomatics University of Cape Town Cape Town South Africa; ^26^ Department of Organisms and Systems Biology (Ecology Unit) and Research Unit of Biodiversity (UO‐CSIC‐PA) University of Oviedo Oviedo Mieres Spain; ^27^ U.S. Geological Survey Western Geographic Science Center Moffett Field California USA; ^28^ School of Science and the Environment University of Worcester Worcester UK

**Keywords:** Canopy height model, drone, fine spatial resolution remote sensing, plant height, structure‐from‐motion photogrammetry, UAV

## Abstract

Non‐forest ecosystems, dominated by shrubs, grasses and herbaceous plants, provide ecosystem services including carbon sequestration and forage for grazing, and are highly sensitive to climatic changes. Yet these ecosystems are poorly represented in remotely sensed biomass products and are undersampled by in situ monitoring. Current global change threats emphasize the need for new tools to capture biomass change in non‐forest ecosystems at appropriate scales. Here we developed and deployed a new protocol for photogrammetric height using unoccupied aerial vehicle (UAV) images to test its capability for delivering standardized measurements of biomass across a globally distributed field experiment. We assessed whether canopy height inferred from UAV photogrammetry allows the prediction of aboveground biomass (AGB) across low‐stature plant species by conducting 38 photogrammetric surveys over 741 harvested plots to sample 50 species. We found mean canopy height was strongly predictive of AGB across species, with a median adjusted *R* ^2^ of 0.87 (ranging from 0.46 to 0.99) and median prediction error from leave‐one‐out cross‐validation of 3.9%. Biomass per‐unit‐of‐height was similar *within* but different *among,* plant functional types. We found that photogrammetric reconstructions of canopy height were sensitive to wind speed but not sun elevation during surveys. We demonstrated that our photogrammetric approach produced generalizable measurements across growth forms and environmental settings and yielded accuracies as good as those obtained from in situ approaches. We demonstrate that using a standardized approach for UAV photogrammetry can deliver accurate AGB estimates across a wide range of dynamic and heterogeneous ecosystems. Many academic and land management institutions have the technical capacity to deploy these approaches over extents of 1–10 ha^−1^. Photogrammetric approaches could provide much‐needed information required to calibrate and validate the vegetation models and satellite‐derived biomass products that are essential to understand vulnerable and understudied non‐forested ecosystems around the globe.

## Graphical abstract

Working at sites across the globe, we used a standardized protocol to collect and analyse drone data in order to measure the size of many different plants in non‐forest ecosystems. These measurements of canopy height allowed the prediction of aboveground biomass and carbon storage of different plants accurately across the landscape. This new approach to measuring plants enables detailed monitoring of vegetation dynamics and responses to differences in climate or disturbance that can help us understand the changes happening in important and vulnerable non‐forest ecosystems around the world.

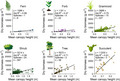

## Introduction

Non‐forest ecosystems, dominated by shrubs and herbaceous plants, cover about 70% of the Earth’s land surface (Duncanson et al., 2019) and account for around 35% of all aboveground biomass (AGB) (Liu et al., 2015). They provide multiple ecosystem services, with critical roles in grazing and agriculture (Asner et al., 2004) and dominate the long‐term trends and inter‐annual variability of the global carbon cycle (Ahlström et al., 2015; Poulter et al., 2014). Understanding the roles these ecosystems play in climate change mitigation and sustainable food production requires information on biomass dynamics (Bartsch et al., 2020; Griscom et al., 2017; Harper et al., 2018). However, monitoring biomass with in situ measurements is labour intensive and thus prone to undersampling, particularly in ecosystems that are spatially heterogeneous and/or temporally dynamic, gaining or losing biomass rapidly (Bartsch et al., 2020; Duncanson et al., 2019; Huenneke et al., 2001; Schimel et al., 2015; Shriver, 2016). Grassland, shrubland, Arctic tundra, savanna and proglacial montane landscapes are often more sensitive to climatic changes than forests (Myers‐Smith et al., 2020) but have received less systematic research attention (Duncanson et al., 2019; McNicol et al., 2018; Sleeter et al., 2018). Gaps in available observations mean that biomass dynamics are not being quantified in many key ecosystems across the globe, hindering the calibration and validation of vegetation models and biomass products derived from satellite observations (Bartsch et al., 2020; Brandt et al., 2018; McNicol et al., 2018). The lack of accurate biomass data limits our ability to track changes and predict future responses in globally important non‐forest ecosystems.

Improving the accuracy of biomass data in non‐forest biomes requires approaches that are sensitive to small absolute differences in AGB, sufficiently inexpensive to be adopted worldwide, and conducive to spatially continuous sampling across representative areas at temporal frequencies appropriate for dynamic ecosystems (Bartsch et al., 2020; Sankey et al., 2021; Shriver, 2016). Non‐destructive estimates of AGB are conventionally obtained from in situ measurements of attributes such as plant cover, height and stem diameters, using functions fitted to harvested biomass observations (Paul et al., 2016; Rudgers et al., 2019). Canopy volume, the product of height and cover, is often the strongest predictor of AGB for low‐stature plants like shrubs and herbs (Alonzo et al., 2020; Bendig et al., 2014; Cunliffe et al., 2020a; Grüner et al., 2019, 2020, 2021; Huenneke et al., 2001; Kröhnert et al., 2018; Schulze‐Brüninghoff et al., 2020; Wijesingha et al., 2019). Remote sensing approaches have been widely used to extend the coverage of biomass predictions. Biomass estimated from spectral reflectance is often highly uncertain due to asymptotic relationships between AGB and surface reflectance and variable soil albedo (Cunliffe et al., 2020a; Myers‐Smith et al., 2020). Biomass can be predicted from airborne light detection and ranging (LiDAR) in shrublands and savannas (Greaves, 2017) but the footprints sampled by LiDAR can be insensitive to fine‐scale changes in plant structure and these data are prohibitively expensive in many areas. Globally available biomass products from space‐based sensors such as LiDAR, synthetic‐aperture radar or vegetation optical depth are either insensitive and/or poorly calibrated and validated in low biomass (<20 Mg ha^−1^) ecosystems (Bartsch et al., 2020; Brandt et al., 2018; Dubayah et al., 2020; Duncanson et al., 2019; McNicol et al., 2018; Sleeter et al., 2018).

Photogrammetry products derived from overlapping aerial images acquired with unoccupied aerial vehicles (UAVs, often referred to as ‘drones’ Joyce et al., 2021) could greatly improve the quantification of AGB in non‐forest ecosystems. This is true for (a) direct cases at local scales and (b) indirectly through enabling improvements in the calibration and validation of biomass products obtained from satellite observations over larger extents. Advances in photogrammetry, particularly structure‐from‐motion (SfM) with multi‐view stereopsis (Westoby et al., 2012), have made it possible to capture 3D representations of plants, quantitatively describing fine‐scale structures (Cunliffe et al., 2016; Dandois & Ellis, 2013; Wallace et al., 2017). SfM allows objective measurements of canopy height at sub‐decimetre spatial grain for a wide range of plants (Bendig et al., 2014; Cunliffe et al., 2016; Frey et al., 2018; Grüner et al., 2019; Kröhnert et al., 2018; Lussem et al., 2019; Poley & McDermid, 2020; Wallace et al., 2017). Lightweight and inexpensive UAV surveys can capture detailed coverage of 1–10 ha extents that are more representative than manual sampling in heterogeneous ecosystems (Cunliffe et al., 2016; Huenneke et al., 2001) and enable spatially explicit comparisons with other observations (Bartsch et al., 2020; Bouvet et al., 2018; Cunliffe et al., 2020b; Duncanson et al., 2019) at temporal intervals appropriate for highly dynamic ecosystems (Bouvet et al., 2018; Cunliffe et al., 2019; Jeziorska, 2019; Shriver, 2016; Yang et al., 2020). Several studies have indicated that UAV‐based survey approaches have the potential to address this observation gap; however, differences in collection, processing and analysis of UAV data between groups prevent cross‐site synthesis and have impeded progress in this field.

Fully realizing the potential of UAV photogrammetry in plant science requires reproducible workflows that minimize biases (Cunliffe & Anderson, 2019; Frey et al., 2018; Tmušić et al., 2020). Furthermore, effective application of UAV photogrammetry to plant science requires knowledge of the relationships between photogrammetry‐derived canopy height and AGB across a range of plants and ecosystems, as well as the systematic understanding of the possible influences of environmental conditions (Cunliffe et al., 2016; Dandois et al., 2015; Frey et al., 2018; Kröhnert et al., 2018; Pätzig et al., 2020). In particular, wind speed may influence retrieved canopy metrics by causing movement of the foliage between image capture and sun angle may influence retrieved canopy metrics by altering the distribution of shadows on different parts of canopies (Dandois et al., 2015; Frey et al., 2018), yet their overall effects are poorly understood in operational contexts. Thousands of hectares of low stature ecosystems have been surveyed with UAVs across the globe over recent years, yielding information‐rich datasets. However, UAV‐photogrammetry products are sensitive to the ways in which data are (i) collected (e.g. ground sampling distance, image overlap, viewing geometry, spatial control, illumination conditions) (Cunliffe et al., 2016; Dandois et al., 2015; Frey et al., 2018; James et al., 2020; James & Robson, 2014; Mosbrucker et al., 2017; Tmušić et al., 2020), (ii) processed (e.g. software, lens model, control accuracy, processing quality, depth filtering) (Cunliffe et al., 2016; James et al., 2020; James & Robson, 2014; Mosbrucker et al., 2017) and (iii) analysed (e.g. canopy height metrics, spatial grain and interpolation method, statistical treatment) (Cunliffe et al., 2016, 2020b; Grüner et al., 2019; Lussem et al., 2019; Poley & McDermid, 2020; Wallace et al., 2017). These sensitivities are more pronounced for subjects with complex texture, such as vegetation, and hinder comparisons between measurements obtained from different workflows. Maximizing the value of photogrammetric approaches for ecological insight, therefore, needs standardized and reproducible protocols (Cunliffe et al., 2020b; Pätzig et al., 2020) but few efforts currently exist to advance this aim.

In this study, we tested the capacity of a new UAV data collection and analysis protocol to deliver standardized measurements for allometric inference of biomass in non‐forested ecosystems globally. We asked the following research questions: (1) Does canopy height derived from UAV photogrammetry correspond with AGB at the species level? (2) Does photogrammetry‐derived canopy height correspond with AGB at the plant functional type (PFT) level? (3) Are the relationships between reconstructed canopy height and biomass influenced by wind speed and (4) solar elevation?

## Materials and Methods

### Sampling design

**Figure 1:**
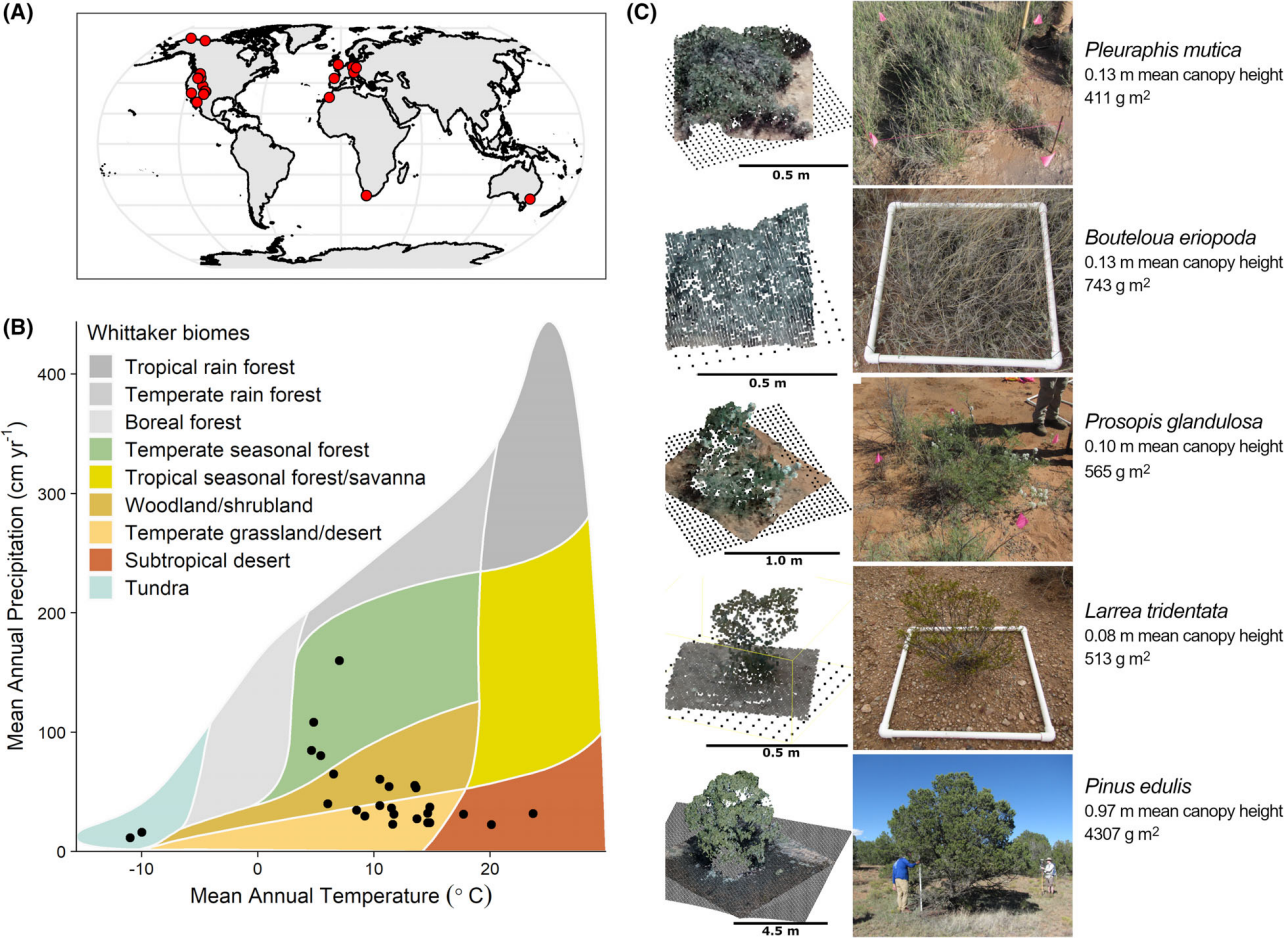
Point clouds derived from UAV surveys provided structural reconstructions of plants across globally distributed non‐forested ecosystems. Our sampling across four continents (A) encompassed five bioclimatic zones where low stature vegetation is often dominant, representing most of the non‐forest biomes described by Whitaker (1975) (B). Reconstructed point clouds with grid of black points representing the modelled terrain correspond strongly with photographs of harvest plots (C).

We invited over 400 researchers from remote sensing, UAV photogrammetry and vegetation science communities around the world to participate in this experiment and collect new data using the same rigorous field protocol (Cunliffe & Anderson, 2019). Our field protocol was informed by a large body of previous work (Assmann et al., 2018; Cunliffe et al., 2016, 2020a, 2020b; Dandois & Ellis, 2013; Duffy et al., 2017; Frey et al., 2018, etc.) and was designed to deliver comparable datasets across different users using different tools and working in different ecosystems. We focused on sampling low stature phenotypes across a diverse range of non‐forest ecosystems, including Arctic tundra, woody savanna, proglacial montane and semi‐arid and temperate grassland and shrubland sites (Fig. 1B). We asked participants to select target species that were regionally widespread, accessible and would inform ongoing research efforts. Sampling was undertaken during seasonal peak canopy cover to try to minimize differences due to phenophase.

### Aerial imaging surveys

We used lightweight UAVs to capture aerial visible (red‐green‐blue) photographs of each harvest site (see Table S1 for details on each site and camera). For each site, two sets of survey flights were undertaken, the first acquiring nadir images with a spatial grain of ca. 5 mm per pixel at the canopy top, and the second acquiring oblique (ca. 20° from nadir) images from ca. 4‐m higher altitude. Survey altitudes, therefore, varied depending on the spatial resolution and field‐of‐view of the sensors and the canopy height but were typically ca. 20 m above the canopy. The different perspectives afforded by the nadir and higher, convergent surveys improved the camera network stability (Aber et al., 2019; Hendrickx et al., 2019; James & Robson, 2014; James et al., 2017; Luhmann et al., 2016; Mosbrucker et al., 2017; Nesbit & Hugenholtz, 2019). Each survey obtained 75% forward and side overlap, together capturing at least 30 images for each part of the study area. High image overlap facilitated tie point matching in vegetated scenes. Wind speeds were generally recorded using handheld anemometers ca. 2 m above ground level immediately prior to the survey (Duffy et al., 2017). Our sampling protocol (Cunliffe & Anderson, 2019) was optimized for smaller plants of up to ca. 3 m in height (see Note S1 for further discussion). A key requirement for photogrammetric surveys is the inclusion of adequate spatial control (Aber et al., 2019; James et al., 2020). We used thirteen ground markers, deployed across each site and geolocated to a typical precision of ±0.015 m horizontally and ±0.03 m vertically to constrain our reconstructions.

### Vegetation harvests

We sampled a total of 741 harvest plots with AGB ranging from 9 to 7892 g m^−2^, mean canopy heights ranging from 0 to 1.9 m and maximum canopy heights ranging from 0.01 to 6.7 m. We used an area‐based approach to enable sampling in ecosystems with continuous or coalesced canopies, while also sampling individual plants where these were naturally isolated from other plants (Cunliffe & Anderson, 2019; Cunliffe et al., 2020b). We selected harvest plots to sample across the natural range of canopy heights observed at each site in order to efficiently estimate the allometric models and assess the form of the relationship between mean canopy height and biomass (Warton et al., 2006). Plots were chosen to try to ensure that ≥90% of the biomass and ≥90% of the foliar volume within each plot was associated with the target species. The protocol detailed that sampling plots should be a minimum size of 0.5 × 0.5 m to minimize the effects of co‐registration errors. The corners of each plot were geolocated with a high‐precision global navigation satellite system (GNSS) before all standing biomass was harvested to ground level (or the moss level for *Salix richardsonii* and *Arctophila fulva*) (Cunliffe et al., 2020a). Biomass was then dried at ca. 50–80°C until reaching a constant weight over a 24‐h period. For the largest taxa (*Adenostoma fasciculatum, Adenostoma sparsifolium, Atriplex polycarpa, Ericameria nauseosa, Juniperus monosperma, Launaea arborescens, Pinus edulis* and *Prosopis velutina*), freshly harvested biomass was immediately weighed in the field and representative sub‐samples were dried to determine moisture contents (Cunliffe et al., 2020b).

### Image‐based modelling

Aerial images were processed using SfM photogrammetry, using well‐established workflows that have been shown to deliver accurate results in low stature ecosystems (Cunliffe et al., 2020a, 2020b; Cunliffe et al., 2016). Geotagged image data and ground‐control marker coordinates were imported into AgiSoft PhotoScan Professional v1.4.3 (now Metashape, http://www.agisoft.com) and converted to UTM coordinate reference systems. Image sharpness was measured using PhotoScan’s image quality tool, all images had image sharpness scores ≥0.5 (Mosbrucker et al., 2017). Interior (lens distortion) and exterior (position and orientation) camera parameters were estimated using PhotoScan’s highest quality setting, a key point limit of 40 000, a tie point limit of 8000, with generic and reference pair preselection enabled, and adaptive camera model fitting disabled. During camera self‐calibration, we estimated focal length (f), principal point (cx, cy), radial distortion (k1, k2), tangential distortion (p1, p2), aspect ratio and skew coefficient (b1, b2) lens parameters. Most cameras had global shutters but rolling shutter corrections were used when appropriate. Reference parameters were set to the following: camera location accuracy = *XY* ± 20 m, *Z* ± 50 m; marker location accuracy = *XY* ± 0.02 m, *Z* ± 0.05 m; marker projection accuracy was set to 2 pixels; tie point accuracy was set to either the mean root mean square reprojection error or one, whichever was greater. Camera alignment produced a sparse point cloud that was then filtered to exclude points with reprojection error >0.45 pixels. The sparse point clouds and estimated camera positions were reviewed for plausibility, and any obviously erroneous tie points or cameras were removed manually. Digital markers were placed by an operator on 10 projected images for each of the 13 ground markers. Ten of these markers were used to constrain the photogrammetric reconstructions spatially (Ribeiro‐Gomes et al., 2016), while the remaining three used for accuracy assessment were deselected before the camera parameters were optimized. Any obviously implausible camera positions were refined after marker placement and optimization. All cameras were aligned in most cases and used for multi‐view stereopsis (dense point cloud generation), using the ultrahigh‐quality setting with mild depth filtering to preserve finer details of the vegetation (Cunliffe et al., 2016, 2020a; Frey et al., 2018; Lussem et al., 2019). For further discussion, see Note S1.

### Terrain modelling

An essential requirement for deriving canopy height models from photogrammetry‐derived point clouds is a digital terrain model, which must be sufficiently accurate and detailed with respect to canopy heights and topographic complexity (Poley & McDermid, 2020). We used terrain models interpolated with Delaunay triangulation between the GNSS‐observed harvest plot corners (Fig. 1C). In instances where plant canopies are discontinuous in space, suitable terrain models could be extracted from the photogrammetric point cloud (Cunliffe et al., 2016; Grüner et al., 2019). Other options include extracting terrain models from photogrammetric UAV surveys during leaf‐off conditions, LiDAR surveys (Wilke et al., 2019) or walkover surveys with GNSS instruments (Cunliffe et al., 2020b).

### Calculation of canopy heights

Point clouds were analysed with PDAL (v2.1.0) (PDAL Contributors, 2020). The point cloud representing each harvest plot was a subset using the GNSS‐observed corner coordinates. In a few instances where plot infrastructure (e.g. marker flags) was visible in the point cloud (*n* = 20 plots), these points were manually classified as noise and excluded from canopy height calculations. Within each plot, the height‐above‐ground of each point was calculated relative to the terrain model and any points with negative heights‐above‐ground were set to zero (Cunliffe et al., 2016; Grüner et al., 2019). Using a 0.01 m resolution grid, we calculated the maximum point height in each grid cell. For cells containing no points, we interpolated heights using inverse distance weighting considering an array of 7 × 7 cells with a power of one, and cells with no points in that neighbourhood remained empty. Plot‐level mean canopy height was then extracted from this grid of local maxima elevations. Mean canopy height, sampled at fine (centimetre) spatial grain, integrates canopy cover and height, as well as foliage density; the consideration of these multiple plant size attributes was fundamental to the robust prediction of biomass using this approach.

### Statistical analysis

Sun elevations were computed with the Astral package (Kennedy, 2020). Statistical analyses were conducted in R (v3.6.1, R Core Team, 2019). Figure 1B was produced using the plotbiomes package (Stefan, 2018). We excluded 13 bryophyte plots from two rocky sites where we were unable to extract meaningful canopy height observations (Fig. S5) and 16 graminoid plots from one grassland site (‘WSP’) that could not be reconstructed (Fig. S6, Note S1).

We used ordinary least squares regression to fit linear models predicting AGB observations from mean canopy height for each plant functional type (PFT) and for each species with four or more observations. We considered ferns, forbs, graminoids, shrubs, trees and succulents as PFTs and constrained the y‐intercept to zero in order to ensure zero canopy height predicted zero biomass. Model performance was validated using leave‐one‐out cross‐validation (LOOCV) to compute the mean out‐of‐sample prediction error, which was divided by the model slope to obtain relative errors for each model (Alfons, 2015; Poley & McDermid, 2020).

**Figure 2:**
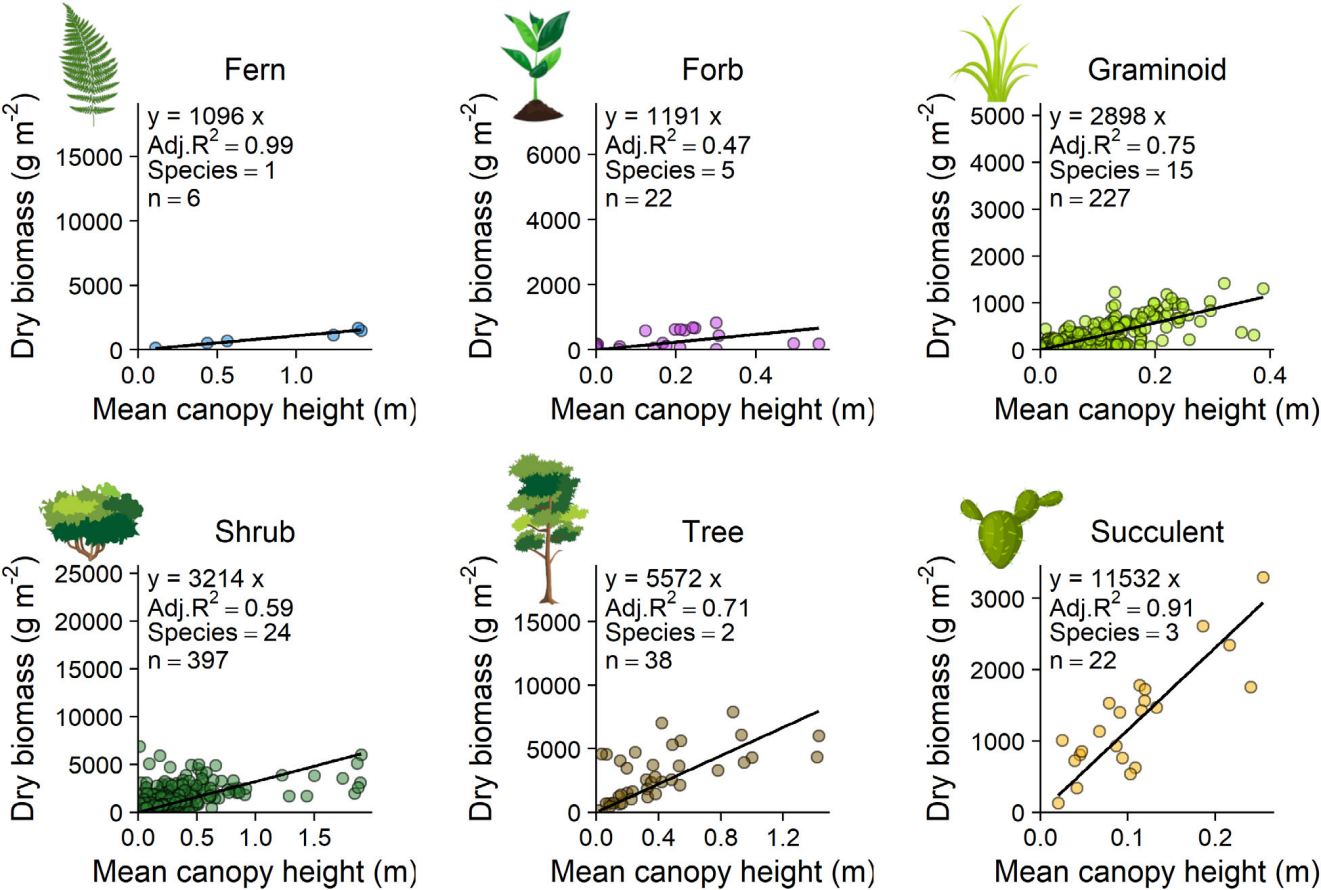
Photogrammetrically derived canopy height was a strong predictor of biomass within most plant functional types. A constant *X*:*Y* ratio was used for all plots, enabling visual comparisons of model slopes even though axis ranges vary. Model slopes were generally similar within but differed between, plant functional types. ‘Species’ indicates the number of species pooled for each plant functional type and black lines are linear models with intercepts constrained through the origin. Full model results are included in Table 1.

**Figure 3:**
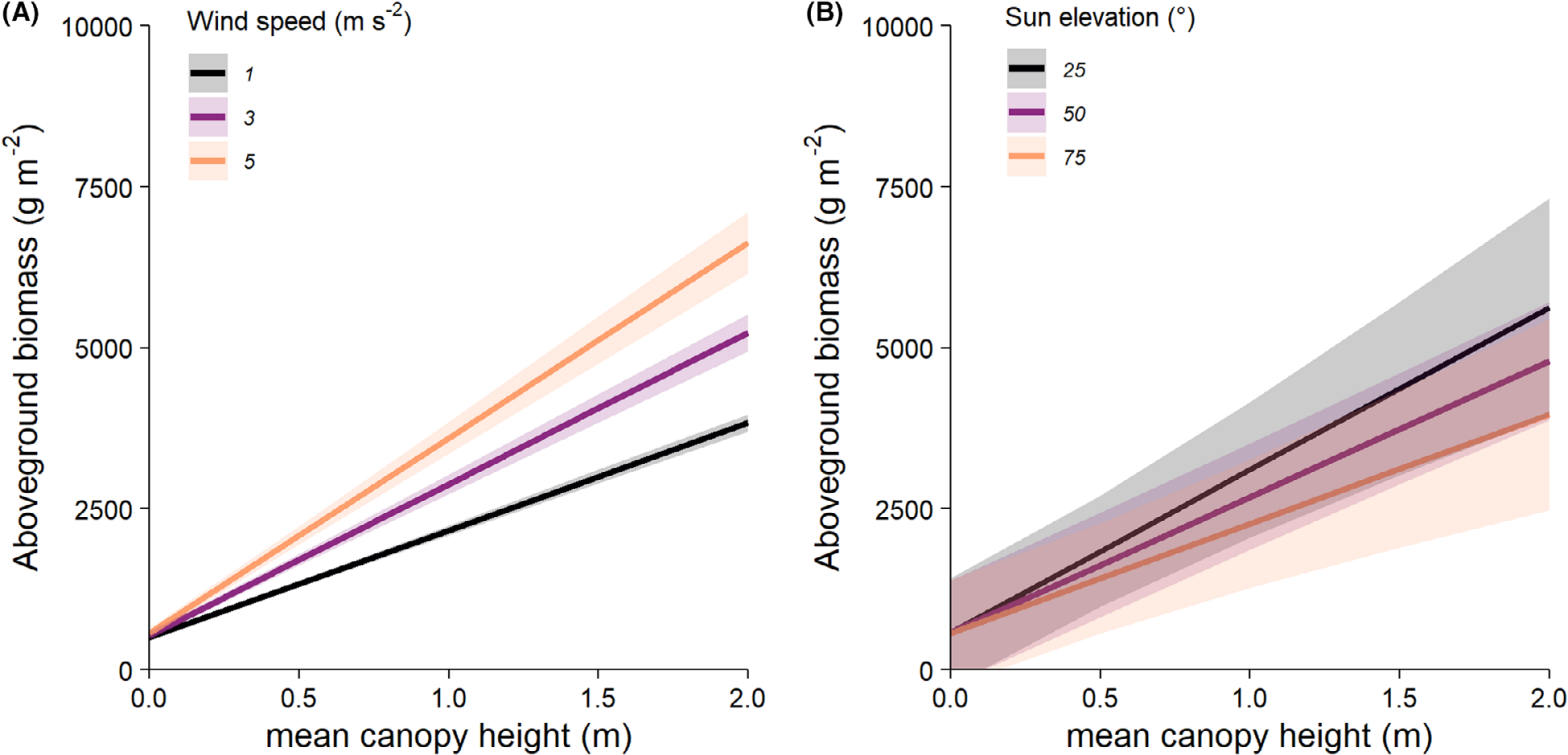
Reconstructed plant height and thus height–biomass relationships were systematically influenced by near‐ground wind speed but were insensitive to sun elevation. Mean predicted aboveground biomass variation over the range of observed mean canopy height, estimated for a range of three wind speeds and sun elevations. Wind speed had a statistically clear and positive effect on the relationship between height and biomass (A) (Figs. S2A and S3, Table S3) but sun elevation had no significant effect on the relationship between height and biomass (B) (Figs. S2B and S5, Table S5). Shaded areas represent 95% confidence intervals on the model predictions.

To test whether near‐ground wind speed influences allometric functions, we fitted a generalized linear mixed model (GLMM) to predict total biomass as a function of canopy height and wind speed as fixed effects and PFT as a random effect based on a gamma error distribution with an identity link function, using the ‘lme4’ package (v1.1–23) (Bates et al., 2015) (Table S3). Succulents were excluded because their inclusion prevented model convergence, possibly because this PFT had a much steeper slope between height: biomass (Table 1, Fig. 2) and/or because they may be less influenced by wind speed (Fig. S2). To illustrate the effect of wind speed, we used the ‘ggeffects’ package (v0.15) (Lüdecke & Aust, 2020) to simulate the relationship between height and biomass for three levels of wind speed using the GLMM (Fig. 3A), and plotted the slope of biomass–height models (±83% confidence interval (Krzywinski & Altman, 2013)) against wind speed at the PFT (Fig. S2A) and species levels (Fig. S3). There was insufficient replication to allow convergence of more complex model structures including species nested within PFT or site as random effects. We evaluated diagnostics for all models visually using the R package ‘performance’ (v0.4.6) (Lüdecke et al., 2020).

**Table 1:** Parameters for linear models fitted to each plant functional type. LOOCV is the prediction error from Leave‐One‐Out Cross‐Validation divided by the slope.

Plant functional type	*n*	*n* of surveys	Slope g m^−2^	Residual standard error g m^−2^	Adj. *R* ^2^	*t*‐statistic	*P* value	LOOCV %
Fern	6	1	1096	53	0.99	20.558	<0.0001	12.0
Forb	22	3	1191	262	0.47	4.534	0.0002	19.0
Graminoid	227	17	2898	112	0.75	25.786	<0.0001	3.7
Shrub	397	24	3214	134	0.59	23.823	<0.0001	11.6
Tree	38	2	5572	577	0.71	9.654	<0.0001	16.7
Succulent	22	3	11 532	760	0.91	15.159	<0.0001	2.6

To test whether cloud cover influenced allometric functions, we fitted a linear mixed model (LMM) to predict total biomass as a function of canopy height, with PFT as a random effect and cloud cover as fixed effects, using the ‘lmerTest’ package (v3.1–2) (Kuznetsova et al., 2020) (Table S4). Cloud cover was coded as a binary factor, with relatively clear sky (*n* = 620) and cloudy conditions where the sun was obscured (*n* = 80, sky codes ≥6 after Assmann et al., 2018, Table S6). To illustrate the effect of cloud cover, we simulated the modelled relationship between height and biomass for the two levels of cloud cover using the LMM (Fig. S4).

To test whether sun elevation influences allometric functions, we fitted a LMM to predict total biomass as a function of canopy height and sun elevation as fixed effects and PFT as a random effect, using the ‘lmerTest’ package (v3.1–2) (Kuznetsova et al., 2020) (Table S5). We only included observations collected under relatively clear sky conditions (*n* = 620, sky codes ≤5). To illustrate the effect of sun elevation, we simulated the modelled relationship between height and biomass for three levels of sun elevation using the LMM (Fig. 3B), and plotted the slope of biomass–height models (±83% confidence interval, after Krzywinski & Altman, 2013) against sun elevation at the PFT (Fig. S3B) and species level (Fig. S5).

## Results

### Coordinated sampling across a new global network

In response to our request for collaboration, researchers across 28 institutions collected and shared data using consistent data collection protocols (Cunliffe & Anderson, 2019). We sampled 36 sites with a global distribution spanning from 71° North to 37° South, across North America, Europe, Australia and Africa (Fig. 1) and from sea level up to 2800 m AMSL. Two sites were sampled in consecutive years, giving 38 surveys from 36 sites (Table S1). Across our new global network, we sampled 50 low stature plant species across six PFTs including ferns, forbs, graminoids, shrubs, succulents and trees that covered phylogenetic diversity including non‐flowering plants and the most species‐rich clades of flowering plants (including monocots and eudicots), representing the first such coordinated photogrammetric ecological experiment of its kind. While our field measurements did not constitute a random or systematic sample, they did encompass a broad range of plant communities.

### Height–biomass relationship at the species level

Photogrammetrically measured mean canopy height was strongly predictive of AGB at the species level. Linear models with a zero intercept provided good approximations of the relationships between mean canopy height and AGB and are readily interpreted (Fig. 2 and Fig. S1) (Cunliffe et al., 2020a; Poley & McDermid, 2020). The slopes from these models are equivalent to AGB density (g m^−3^, calculated by dividing g m^−2^ by mean canopy height). Species‐level densities ranged between 375 and 13 801 g m^−3^ (Fig. S1, Table S2). Mean canopy height was an accurate predictor for individual species, especially when calibrated for specific ecophenotypic and phenological conditions (Huenneke et al., 2001; Poley & McDermid, 2020; Rudgers et al., 2019). Model goodness‐of‐fit was strong, with adjusted *R* ^2^ values ranging from 0.46 to 0.99 and a median of 0.87 (Fig. S1, Table S2). Leave‐one‐out cross‐validation indicated a median prediction error of 3.9% (Table S2). The carefully designed standardized protocol (Cunliffe & Anderson, 2019) for data acquisition and processing yielded a good level of success in reconstructing 93% (688 out of 741) of harvest plots (Fig. 1C). The few instances where reconstructions were unsuccessful (including mosses in rocky terrain, tall and dense grassland, and plants mostly taller than >3 m) are discussed further in Note S1. The similarities of the height–biomass relationships (Table 1 and Table S2) indicate this approach was generalizable across growth forms and environmental settings.

### Height–biomass relationship at the PFT level

Relationships between height and biomass were similar within plant functional types. For every 1‐cm increase in mean canopy height, AGB increased by 11–115 g m^−2^, depending on PFT (Fig. 2, Table 1). Adjusted *R* ^2^ ranged from 0.49 to 0.99 (Fig. 2, Table 1). Ferns had the lowest density (1096 g m^−3^), followed by forbs (1191 g m^−3^), then graminoids (2898 g m^−3^) and shrubs (3214 g m^−3^) with similar densities, then small trees (5572 g m^−3^) and lastly succulents with the greatest density (11 532 g m^−3^). Species‐level model slopes were generally similar *within* but different *between,* PFTs. Should resource limitations or taxon conservation status preclude destructive harvests for local calibrations, the height–mass models described here could be used to estimate AGB from similar UAV‐derived canopy height models (Table 1 and Table S2). These allometric relationships were linear across the range of canopy height and biomass that we sampled, allowing their application from the whole plant level to the ecosystem level without necessarily requiring the discrete analysis of individual plants that can be challenging in ecosystems with coalesced canopies.

### Influence of wind speed and illumination conditions on reconstructed canopy height

Wind speed negatively affects canopy heights reconstructed from photogrammetry (Fig. 3A, Figs. S2 and S3, Table S3). We found the height‐wind interaction parameter was strong and highly significant (*P* < 0.0001) (Fig. 3A, Table S3). This influence was seen at both the PFT level (Fig. S2A) and species level (Fig. S3). Biomass divided by height increased for surveys conducted in windier conditions because foliage movement meant lower mean canopy heights were reconstructed from images that were acquired non‐concurrently (see Data S2 for an extended discussion). However, the wind had only limited influence on our study because most of our surveys were conducted in relatively light wind conditions (of <3 m s^−1^) (Table S1). We expect sensitivity to wind speed differs between species because the effects of wind on foliage motion depend on canopy architecture and mechanical properties like limb stiffness (Fig. S3, Note S2). Sun elevation had no detectable effect on reconstructed plant height (Fig. 3B, Figs. S2B and S5, Table S5). Cloudy conditions appeared to affect allometric density; however, we considered this finding unreliable due to the imbalance in observations under cloudy and clear conditions (*n* = 80 vs. *n* = 620, respectively, Table S4, Fig. S4). Our study demonstrates the need to control the influence of wind speed in future work particularly when conducting fine‐scale surveys of low stature plants. Surveying under low wind speeds may be a higher priority than optimal (near‐nadir) solar elevations for obtaining reproducible structural models of vegetation.

## Discussion

Using a newly developed photogrammetric data collection protocol, we were able to measure structural plant traits across a globally distributed set of low stature ecosystems. Comparable data collection by participants from 28 institutes across 50 non‐forest plant species enabled us to establish and compare height–biomass relationships. Our sample achieved a more than 20‐fold improvement in the coverage of harvest plots, species and sites compared to existing photogrammetry vegetation studies (Fig. 1C) (Grüner et al., 2019; Lussem et al., 2019; Wallace et al., 2017). The relationships between canopy height and biomass appeared linear at the species and PFT levels across a diverse range of low stature ecosystems (Fig. 2 and Fig. S1). Linear allometric functions can be applied from the whole plant level to the ecosystem level without necessarily requiring the discrete analysis of individual plants that can be challenging in ecosystems with coalesced canopies (Bartsch et al., 2020; Cunliffe et al., 2016; Krofcheck et al., 2016; Poley & McDermid, 2020).

The high goodness‐of‐fits and low average prediction errors (Table 1 and Table S2) indicate accuracy was as good as conventional in situ allometric approaches reported in the literature (Chieppa et al., 2020; Cunliffe et al., 2020b; Huenneke et al., 2001; Muldavin et al., 2008; Rudgers et al., 2019). Species‐level model slopes were generally similar *within* but different *among,* PFTs, indicating these relationships appear generally transferrable between species within PFTs (Chieppa et al., 2020; Paul et al., 2016), particularly for the better‐sampled types such as graminoids and shrubs, although phenotypic and phenological variation will always limit accuracy (Paul et al., 2016; Poley & McDermid, 2020; Rudgers et al., 2019). While UAV photogrammetry certainly can be used to characterize forest canopies (Dandois et al., 2015; Frey et al., 2018; Poley & McDermid, 2020), tree‐dominated forest ecosystems are often better candidates for measurement with other remote sensing approaches such as LiDAR (Dubayah et al., 2020; Duncanson et al., 2019; Herold et al., 2019), synthetic‐aperture radar (McNicol et al., 2018) or vegetation optical depth (Brandt et al., 2018; Rodríguez‐Fernández et al., 2018; Tian et al., 2016). The similarity of graminoid and shrub PFT relationships indicates these could be applied together to estimate AGB in some mixed ecosystems, without the need to individually classify these types, although allometric functions may need to be calibrated locally in some cases (see Note S4 for further discussion). The LOOCV prediction errors were sensitive to the number of subsamples (e.g. surveys and/or species) sampled for each taxon and they should therefore be compared carefully between taxonomic groups.

Our findings show that our designed protocol enables observations that provide new insights into ecosystem dynamics at previously understudied scales across non‐forested ecosystems. Other groups following this now proven workflow will be able to further extend this understanding to a greater range of ecosystems species and environmental conditions in the future. Mean canopy height is readily compared between taxa, ecosystems and observation approaches (Bartsch et al., 2020; Cunliffe et al., 2020a), so these linear relationships are straightforward to interpret (Warton et al., 2006) and can be easily integrated with landscape modelling frameworks. UAV photogrammetry is well suited for local‐scale observation in non‐forest ecosystems. Intensive UAV surveys are relatively easy to conduct over larger spatial extents of several hectares, which are critical to advancing beyond existing in situ approaches and bridging the scale gap between on‐the‐ground monitoring and the coarser grain of global‐scale products derived from satellite‐based remote sensing (Cunliffe et al., 2016; Poley & McDermid, 2020). Accurate information at these intermediary scales is invaluable for validating models and testing the scaling of ecological relationships and biomass carbon estimates from plots to biomes (Alonzo et al., 2020; Bartsch et al., 2020; Cunliffe et al., 2020a; Myers‐Smith et al., 2020).

Addressing critical knowledge gaps in plant science with UAV photogrammetry demands standardized protocols because photogrammetry‐derived models are sensitive to the ways in which data are collected (Cunliffe et al., 2016; Dandois et al., 2015; Frey et al., 2018; James et al., 2020; James & Robson, 2014; Mosbrucker et al., 2017), processed (Cunliffe et al., 2016; James et al., 2020; James & Robson, 2014; Mosbrucker et al., 2017) and analysed (Cunliffe et al., 2016, 2020b; Grüner et al., 2019; Lussem et al., 2019; Wallace et al., 2017). These sensitivities hinder comparisons between products obtained from different workflows and can be more pronounced for subjects with complex texture, such as vegetation. We anticipate ongoing improvements to camera geolocation and orientation information will continue to improve the accuracy and reliability of the camera parameter estimation, particularly in densely vegetated and texturally complex settings (see also Data S1) (Aber et al., 2019; Chudley et al., 2019; James et al., 2020; Tmušić et al., 2020; Zhang et al., 2019). The lack of systematic and reproducible protocols has impeded the use of UAV data in ecological research to date, so we call for the continued development of harmonized and community‐based protocols to maximize knowledge gains and support cross‐biome syntheses (Cunliffe & Anderson, 2019; Pérez‐Harguindeguy et al., 2013; Poley & McDermid, 2020; Tmušić et al., 2020).

Using a standardized protocol allowed us to investigate how wind speed (Fig. 3A) and solar elevation (Fig. 3B) (Dandois et al., 2015) influenced our findings. We found that it was important to account for the effects of wind speed during photogrammetric surveys beyond simply considering how wind affects aircraft performance. A few previous studies reported contradictory effects of wind speed on forest canopy reconstructions (Dandois et al., 2015; Frey et al., 2018) but we think that these findings may be affected by different spatial grains of analysis (Note S2). Previous studies have also reported contradictory effects of sun elevation on forest canopy reconstructions (Dandois et al., 2015; Frey et al., 2018); however, illumination conditions affect photogrammetry in complex ways (Aber et al., 2019; Mosbrucker et al., 2017), with the influence of sun elevation depending on the distribution and intensity of shadows as well as the camera sensor properties and user choices during surveys and processing (see Note S3 for an extended discussion). When comparing findings regarding illumination effects, it is therefore necessary to consider the capabilities of the sensors and workflows employed relative to the observed subject. The most reproducible reconstructions will be obtained under ‘zero’ wind speeds (Dandois et al., 2015; Frey et al., 2018; Mosbrucker et al., 2017) and similar illumination conditions although this is often difficult to achieve under real‐world operational conditions (Aber et al., 2019; Duffy et al., 2017; Poley & McDermid, 2020). Our findings demonstrate that data will be most comparable when near‐ground wind speeds are similar but also that, where differences are unavoidable, it will be possible to derive corrections for how wind influences canopy reconstructions.

## Conclusion

Our findings show UAV photogrammetry can yield informative canopy height models capable of detecting ecologically significant differences in AGB across a diverse range of low stature ecosystems globally. UAVs have considerable advantages as data collection platforms for ecological applications, including their relatively low cost (although see Note S5), versatility in deployment allowing high temporal resolution monitoring and capacity to record fine‐grained and spatially explicit data (Aber et al., 2019; Anderson & Gaston, 2013; Tmušić et al., 2020). Systematic and comparable observations of plant canopy structure and biomass are vital for calibrating and evaluating vegetation models and biomass products retrieved from globally available remote sensing systems (Bouvet et al., 2018; Duncanson et al., 2019; Rodríguez‐Fernández et al., 2018; Tian et al., 2016). UAV data collection can broaden the scope of research and monitoring programmes to obtain more representative observations in vulnerable and understudied low stature ecosystems. Photogrammetric approaches for monitoring canopy height and biomass provide novel tools that should be used more widely by the ecological research community to improve assessments of ecosystem change and global carbon budgets.

## Authors’ Contributions

A.M.C. conceived the research idea, administered the project, curated the data, did the data visualization and led the writing of the paper. A.M.C. and K.A. developed the experimental design. A.M.C., K.A., R.E.B., I.H.M.‐S., M.M.B., P.E.C., M.D.C., J.M.F.‐G., A.G., N.P.H., C.A.H., P.H., J.W.K., I.M., L.A.M.‐B., S.C.P., J.P., S.S., J.S., S.A.V. and M.L.V. acquired funding. A.M.C., K.A., F.B., R.E.B., I.H.M.‐S., T.A., M.M.B., L.C., P.E.C., M.D.C., M.S.E.‐L., S.M.E., J.M.F.‐G., A.G.F., K.G., B.M.G., A.G., N.P.H., M.S.H., S.H., C.A.H., P.H., W.J., J.W.K., M.K., S.K., M.B.L., I.M., M.E.M, C.D.M., D.M., L.A.M.‐B., S.C.P., J.P., E.S.‐A., K.J.S., D.S.‐B., P.S., S.S., J.S., C.S., S.S.‐S., S.A.V., M.L.V., F.V., M.W., H.W. and R.W. undertook the investigation. A.M.C. and H.A.G. developed the processing program. A.M.C., I.H.M.‐S. and H.A.G. performed the analysis. All authors contributed to the final version of the paper.

## Supporting information


**Figure S1**. Photogrammetrically derived canopy height is a strong predictor of biomass across species.
**Figure S2**. Reconstructed plant height and thus height‐biomass relationships were influenced by wind speed but were insensitive to sun elevation.
**Figure S3**. The sensitivity of photogrammetrically reconstructed height to wind speed differs between species based on growth form.
**Figure S4**. The apparently strong effect of cloud cover on photogrammetrically reconstructed height likely arises from imbalanced observations.
**Figure S5**. Sun elevation has little systematic effect on photogrammetrically reconstructed height at the species‐level.
**Figure S6**. This sampling approach was unable to usefully resolve the canopy height of mosses.
**Figure S7**. Image alignment was not possible in this tall grassland, due to the complicated texture and structure of the subject preventing the accurate matching of tie points.
**Table S1**. Details of survey location, climate, ecosystem type and image sensor.
**Table S2**. Parameters for species‐level linear models, fitted for all species with four or more observations.
**Table S3**. Generalised linear mixed model parameters testing wind effects.
**Table S4**. Linear mixed model parameters testing cloud cover effects.
**Table S5**. Linear mixed model parameters testing sun effects.
**Table S6**. Sky Codes for qualitative classification of cloud‐related ambient light conditions.
**Note S1**. Notes on the limitations of photogrammetric reconstructions of plants.
**Note S2**. Notes on how wind speed influences canopy heights.
**Note S3**. Notes on how sun elevation influences canopy heights.
**Note S4**. Limitations on ‘universal’ allometries.
**Note S5**. Notes on costs.

## Data Availability

The data collected for this publication, including aerial images, marker and plot coordinates and dry sample weights, as well as site and survey metadata, are available from the NERC Environmental Information Data Centre <https://doi.org/10.5285/1ec13364‐cbc6‐4ab5‐a147‐45a103853424>. Code for photogrammetric processing and statistical analysis is available at Zenodo <https://doi.org/10.5281/zenodo.4783021>
